# Emerging 21^st^ Century Medical Technologies

**DOI:** 10.12669/pjms.303.5211

**Published:** 2014

**Authors:** Mohammad Bajwa

**Affiliations:** 1 Mohammad Bajwa, PhD, Program Director, Healthcare Systems Management, Metropolitan College of New York, 431 Canal Street, NY 10013, USA.

**Keywords:** Emerging medical technologies, Electronic Health Record (EHR), Electronic Medical Record (EMR), Radio Frequency Identification (RFID), Global Positioning System (GPS), Telemedicine, Smart phones, Tablet Personal Computers, Patient portals, Nanomedicine

## Abstract

Although several medical technologies have been around since decades and are in the continuous process of development, some latest technologies are changing the way medicine would be practiced in the future. These technologies would allow medical practice from anywhere, any time and from any device. These include smart phones, Tablet PCs, Touch screens, digital ink, voice recognition, Electronic Health Records (EHRs), Health Information Exchange (HIE), Nationwide Health Information Network (NwHIN), Personal Health Records (PHRs), patient portals, Nanomedicine, genome-based personalized medicine, Geographical Positioning System (GPS), Radiofrequency Identification (RFID), Telemedicine, clinical decision support (CDS), mobile home healthcare, cloud computing, and social media, to name a few significant.

## INTRODUCTION

Mobile technology is touching virtually every aspect of our lives to the extent of creating dependency. Entry of mobile devices in the healthcare arena is a very recent development and hence is still in infancy. These smart technologies vary from hand held and pocket size devices to iPads that can fit handbags and lap top computers. Their use is not only restricted to healthcare providers, but extended to patients and associated businesses for providing medical care to patients, making appointments, and using health information for billing. Use of mobile technology in the developing countries, with no or little traditional communication infrastructure and in remote areas, for health education, clinical monitoring, and research has been phenomenal. These are just a few applications of the mobile technology in the healthcare with many more and diverse in the pipeline along with their unique challenges, while opening the doors to new opportunities.

The intent of this article is to review these technologies and assess their impact on the 21^st^ Century healthcare.

**SMART PHONES**

Smart phones are smart enough to perform a number of computing activities in addition to the regular phone functions. In fact, smart phones like iPhone and BlackBerry are microcomputers that can access and process host of data and have camera, interfaces, internet browsing, emailing, text and instant messaging, Wi-Fi, and Geographical Positioning System (GPS) capabilities.

Just like they have revolutionized our several facets of life, they have changed the face of healthcare. Some of the ways the smart phones can be and are being used in healthcare both by the providers and the patients are:^1^

**Healthcare Providers:**

- Review patient information.
- Review lab results.
- Provide medical advice.
- Connect to the facility’s electronic health record.
- Create and send prescription to the patient’s Pharmacy.
- As charge captures (capturing procedure and disease codes) and bill patients.
- Make dictation, and
- Manage clinical messages.

**Patients:**

- View, schedule and make appointments using web portals.
- View any medical record from the providers’ web portal.
- View medical information from the Internet.
- Find healthcare providers, and
- Find pharmacies.

**However their use in healthcare has limitations due to:**

- *Interference with medical devices* – smart phones receive and transmit information using radio frequency (RF) and can interfere with some critical medical devices transmitting wired or wireless electric signals such as cardiac monitors and pace makers. Cell phones, however, can be set to *hospital modes* like airplane mode that alleviates such problems.
- *Medical interpretations *– cell phones have small screen size which may conceal some crucial details such as in ECG (Electrocardiography), MRI (Magnetic Resonance Imaging), X-rays, etc.

**TABLET PERSONAL COMPUTERS (Tablet PCs)**

While Tablet PCs have been around for quite a while, healthcare IT applications are relatively recent developments. The use of slate-type tablets like iPad is now becoming very popular in all walks of life including the healthcare. Because of their larger size and wireless capabilities, the Tablets PCs are a balance between screen size and portability. Traditionally Electronic Medical Records (EMR) have been limited to office tables, but with Tablet PCs, users have the ability to access medical records from anywhere like the smart phones.

**The Tablet PCs can allow the healthcare users to:**

- Explain clinical results to patients at the bedside or in the consultation room(s) to enhance discussion of the results and possible courses of treatment by showing these on the Tablet PC in terms of procedures and results.
- Look up resources from anywhere such as drug names, diagnoses and treatment options.
- Consult data just before or during the treatment like surgery or any other type of medical intervention. It can also be attached to barcode scanner, Radio Frequency Identification (RFID) scanner, or camera. Several vendors have already started offering iPad version of EMRs.

**Despite the forgoing benefits of the Tablet PCs, few constraints still remain:**

- Detraction of doctors from the traditional bedside care because the focus may become the Table PC for entering and viewing the information rather than the patient.
- Requires more resource management as the normal PCs are stationary and less like to be moved and misplaced.
- Risk of theft, especially when the Tablets PCs are used to store unencrypted patient sensitive information and they use no authentication as strong password or biometric technology.
- Small screen size still remains a limitation as diagnostic details can remain hidden in ECG, MRI, X-ray, etc. Most diagnostics are made with large screens and multiple monitors to uncover hidden details.

**TOUCH SCREENS**

Touch screens have existed for quite a while in one or the other form but the *resistive* and *capacitive* touch detection has flourished recently allowing pressure sensitivity, multi-touch and gesturing. A touch screen is more institutive to use than mouse. Hence, EMRs/EHRs can benefit from this technology where they can offer:

- *Enhanced usability in hospital settings *where clinicians use gloves or other protective apparel (ER, surgery) or where small number of repetitive tasks need to be done quickly (ER admittance).
- *Innovative user interface* provides rich user experience by touching the icon for the function.
- *Easy navigation and 3D diagnostic models* (cardiac CT scan) can be manipulated with multi-touch rotations.
- *Use at all levels* as the touch screens can be used by even less computer literate persons for some medical functionalities, such as accessing EMR/EHR, lab results, etc.
- They can also be used as patients’ kiosks, and bedside and homecare devices.

**DIGITAL INK**

This technology allows writing on a screen just like on a piece of paper and has been integrated into EMR/EHR. *Microsoft Ink* is one such technology. A charting system can accept stylus input and convert it into text. Its uses in healthcare arena encompass:

- *Drawing images* such as in surgery to communicate between the doctor and the patient.
- *Annotating diagnostic images *such as X-ray and MRI scans to indicate important features.
- *Inputting text naturally*, especially when the patient is unable to communicate and can write a note to the doctor to explain his/her situation.
- *Authenticating reports *quickly and easily by placing signatures just like with wet ink.

**VOICE RECOGNITION**

The normal procedure of medical transcription includes dictating medical notes in some recording device to be translated by an expert transcriber followed by vetting and authentication by the Physician. This can take 1-5 days by the time it becomes a part of the medical record, and sometimes it never does if the Physician does not vet and authenticate the transcribed report. At times the Physician may not even read the report and signs it in hurry leading to incorporation of errors in the medical record.

Voice recognition is one such technology that resolves this issue. The Physician can dictate directly into a computer using microphone that recognizes the spoken words and translates them into text. The Physician can then proofread on screen and digitally sign it. The report can be printed and placed in the medical chart or electronically in EMR/EHR making it a part of the electronic medical record.

**ELECTRONIC MEDICAL RECORD (EMR)/ELECTRONIC HEALTH RECORD (EHR)**

The EMR/EHR is electronic version of the medical record. The basic difference between EMR and EHR is that EMR is restricted to within the facility while the EHR is interlinked between facilities and can be accessed from outside using Internet through wired or wireless technology.

**EHR has several inbuilt capabilities:**

- It can help the physician to view medical record with the touch of button or click of a mouse including medical histories, lab reports, and diagnostic imaging reports.
- Data can be entered directly and becomes a part of the digital medical record.
- Lab results automatically are linked to the patient’s medical record when uploaded to the diagnostic labs Server.
- The inbuilt database of literature helps Physician make evidence-based rather than opinion-based decision, called Clinical Decision Support (CDS) system.
- Reportable medical conditions to the government and the accrediting agencies are transmitted automatically.
- Prescriptions are made by selecting the medicines from the drop down list that avoids illegible handwriting, a major source of medical errors, and transmitted directly to the patients’ pharmacies.

The patients can access their medical records through the patient portals by logging into the provider’s website.

**HEALTH INFORMATION EXCHANGE (HIE) and NATIONWIDE HEALTH INFORMATION NETWORK (NwHIN).**

The next step is linking different EHRs in a region to make a Regional Health Information Organizations (RHIOs) for the exchange of health information in a certain area. Almost every US state now has one or more RHIOs. The RHIOs in turn would be interlinked nationwide to make NwHIN so that the health information could the shared across the nation. Once completed, it will work as healthcare information highway just like the Internet accessible from anywhere, anytime, by any device and anyone authorized.

**PERSONAL HEALTH RECORD (PHR).**

PHR is the latest development in the chain of EHR, HIE and NwHIN which emphasizes the participation of patients more actively in their healthcare decision making. PHR could be paper-based or electronic where the patients would enter data about their health status from different sources. The electronic PHR (ePHR) can be linked to the provider’s EHR for the Physician to review the information compiled by the patient.

**The PHR can lend the following benefits:**

- *Improve patient tracking* – clinicians can monitor disease management, oversee progress, and track medication dosages and compliance.
- *Encourage patient participation *– patients interested in their health take better care of their health. Inputting data into PHR would encourage patients to stay on track with their health maintenance.
- *Offer social networking integration – *through PHR the patients can interact with other patients and share information with those with the same medical issues.

Constraints to PHR include s*ecurity and privacy *of the health information and a*ccuracy and worthiness of data *as the Physician may not trust the data entered by the patients assuming that patients used incorrect technique or unstandardized equipment.

**PATIENT PORTALS**

Patient Portals are healthcare-related online applications that allow patients to interact and communicate with their healthcare providers. Portal services are available on the Internet anytime and from anywhere. Most portal applications are integrated into websites of the healthcare providers or are independent modules linked to the providers’ websites. In either case, patients can access their medical information and interact with providers through the Internet.

**NANOMEDICINE**

Nanomedicine is a science that involves use of minute or miniature nanometer-sized (10^-9 ^of a meter) devices (nanotechnologies) at the level of molecules, slightly above the atomic size of Angstrom (10^-10^ of a meter).

Medical applications of nanotechnologies include imaging the internal organs whereby a small capsule containing nanodevices, such as light source and a camera, is ingested. While passing through the digestive system it emits radio signals that are captured by a receiver worn by patient on a belt around the waste. Nanodevices can also be used in microsurgeries of eyes, blood vessels and in tissue regeneration where they release growth chemicals to catalyze tissue healing. They also have a great potential in controlled release of hormones, enzymes or therapeutic chemicals at the selected sites. They can be designed to be placed under the skin to monitor blood glucose and release insulin accordingly. Their other use could be placement in blood vessels to monitor blood pressure and release of medication to control blood pressure. Efforts are underway to help regenerate neurons (nerves) and brain cells using special nanodevices. Their other significant role would be to repair DNA or replace the defective part of DNA using nanodevices that can carry correct DNA chunk and place at the defective part. Traditionally viruses have been used for this purpose but they have resulted in tumor generation. Nanodevices, being inert, would have huge potential in this regard. Early trials with animals have shown some success.^2^Their use in the genome analysis is also being investigated.

**GENOME-BASED PERSONLAIZED MEDICINE**

Every organism inherits traits from its parents which reside in the chromosomes as DNA segments, called genes. Human genome contains 46 chromosomes, 23 from each parent that house 20-22,000 genes. Each gene pair is responsible for determining one trait. Each male sperm and female ovum contains 23 chromosomes and at the time of fertilization both combine to form 46 chromosomes as zygote that develops first into embryo and then into fetus. From the pair of genes from both the parents responsible for the same trait (e.g. hair color), one becomes dormant to control that trait and the other recessive that stays dormant in the life time of the person.

The offspring is a unique combination of parents’ chromosomes (DNA) and inherits good and bad traits of both the parents. The genome analysis of a person can identify the defective codes that can lead to development of a disease or disorder, called *risk factors*. If genome of a person is known, vulnerability of that person to diseases can be taken care of through prevention and control of the environmental factors that trigger that disease. For example, if a person has messed up BRCA gene, indicative of cancer, he/she should avoid smoking and polluted environments. Similarly, if a person’s diabetic vulnerability is detected, he/she should restrict sugary foods.

DNA expression of traits is greatly affected by the environments. It is often said that *“DNA is a loaded gun but is triggered by the environment”*. *Epigenetics* is the science that researches the effect of environments on the gene expression. The environments that affect gene expression include pollution, radiation, chemicals, and social habits like smoking, drinking, work and food habits.

At present almost 1100 diseases and disorders have been traced back to abnormal genetics due to missing, incorrect, or modified DNA code(s) through *mutations *disorders of DNA that may be inherited or occur during the life time of a person abruptly or over a period. The most common genetic diseases are sickle cell anemia, cystic fibrosis, Duchenne Muscular Dystrophy (DMD), Huntington Disease (HD), breast cancer, prostate cancer, Down syndrome, and several others that owe their origin to error, substitution, addition, deletion, and modification of inherited DNA codes. An effort is in continuum to treat these diseases through stem cells and inserting correct DNA codes for the defective or missing codes.

Some tests are now routinely performed during prenatal and on birth to detect any genetic abnormality and treat or manage it accordingly.

**GEOGRAPHICAL POSITIONING SYSTEM (GPS).**

The Global Positioning System (GPS) is a Global Navigation Satellite System (GNSS). Twenty-four GPS satellites currently orbit Earth and transmit signals to GPS receivers, which determine the location, direction, and speed of the receiver. GPS has multitude of applications in several disciplines. Its main function is to track the location of the signal transmitting unit and has been extensively used in navigation of ships, airplanes and now automobiles. Its use in healthcare is a recent event and is still on the rise.^3^

GPS’s limited use in healthcare includes tracking the location of elderly, especially suffering from Alzheimer’s disease and those living alone; monitoring physical activity like walking; and healthcare research to trace patients. 

**RADIOFREQUENCY IDENTIFICATION (RFID)**

Radio Frequency Identification (RFID) is a fast developing technology that uses radio waves for data collection and transfer; it can capture data efficiently and automatically without human intervention. In the retail supply chain, RFID is already well established as a way to reduce theft and track objects from manufacture through shipment to delivery. For a variety of reasons, adoption of RFID by healthcare has been sluggish because the payback is less immediately visible than what most companies prefer.^4^

RFID tags attached to patients provide identification, tracking, and security. Basic RFID is already being used to track patients for anti-elopement and anti-abduction programs. RFID is also beginning to show use to provide more extensive patient identification than traditional bar coding can, and to track and locate capital equipment within the hospital. In years to come, RFID could be used for a variety of applications, including counterfeiting of medical products.^5^

**TELEMEDICINE**

Telemedicine is the use of medical information exchanged from one site to another via electronic communications to improve a patient’s clinical health status. Telemedicine includes a growing variety of applications and services using two-way video, email, smart phones, wireless tools and other forms of telecommunications technology.^6^ Telemedicine is named after the type of services it renders: telenursing, telepharmacy, telerehabilitation, teleradiology, teletrauma care, telepsychiatry, telepathology, and teledermatology. Major benefits of Telemedicine include improved access to healthcare, cost effectiveness, improved quality and patient demand, especially in rural and remote areas while its constraints are lack of infrastructure and reimbursement from the third-party payers.

**CLINICAL DECISION SUPPORT (CDS)**

Implementation of EHR would have data repositories that would house a vast amount of literature on diagnostics and treatments of diseases and disorders to help Physician to confirm diagnosis and decide the best treatment and method of healthcare delivery. This would shift the emphasis from *opinion-based *medical practice to *evidence-based* medical practice.

**A CDS can:**

- Point a provider to the reference material and information.
- Identify possible risks for adverse events and errors.
- Raise alerts and provide reminders.
- Encourage adherence to standards.
- Analyze clinical performance.
- Perform certain actions.

**Constraints to CDS may include:**

- *Patient data accuracy* – patient data must be entered consistently and accurately for a CDS to contain the latest information.
- *CDS system accuracy* – the CDS system needs to be intelligent enough to be able to identify similar cases and present to the user when requested.
- *Alert fatigue* – a CDS system must not produce too many alerts otherwise user suffers from *alert fatigue* and develops a tendency to ignore.
- *Usability *–CDS systems are often vendor-specific and do not integrate well with other systems and hence the user may choose not to use the CDS when it starts becoming nuisance instead of aid.

Most CDSs are, however, integrated with EMR/EHR systems and harmonize well with its components.

**MOBILE HOME HEALTCHARE**

The mobile home health technology allows patients to communicate current status of their illness to healthcare provider from home. Although still in infancy, its potential for managing chronic illnesses of the aging population is becoming evident. Its existing examples include linkages with blood pressure monitors, glucose monitors, weight scales, pulse oximeters, etc.The existing mobile home healthcare devices are stand alone and require transfer of data to EMR/EHR but future devices will interact with EMRs/EHRs to deliver data directly to the databases.

**CLOUD COMPUTING**

Cloud computing is the use of virtualized servers that can be scaled on demand based on various levels of resource requirements at run-time.^7^ For example, an application running on a cloud can turn on more servers in response to spike in demand for computing resources. Cloud computing also means Application Service Provider (ASP) and Software as a Service (SaaS), where software services are provided to clients through a web for a fee.

The healthcare can benefit from cloud computing in terms of:

- *Scalable hardware* – hardware resources can be turned on based on need.
- *Efficiency and performance* – since servers are virtualized, different instances can reside on the same hardware and also moved around depending on the need to make the best use of hardware without compromising performance.
- *Increased availability*–cloud hardware is fault tolerant and provides 99.9% availability.

**SOCIAL MEDIA**

Social media is the process of people using online tools and platforms to share content and information through conversation and communication. Healthcare organizations are increasingly using social media for communication and information sharing with their customers. The healthcare organizations need a “protective” policy and a program to educate employees and customers about appropriate social media use. Various healthcare organizations may use social media to enhance marketing, branding, recruitment, reputation management, customer relations and customer service.

**Social Media:** ^8^

- Allow a wide variety of content formats (Text, Photos, and Video).
- Are device independent (Computers, Tablets, Mobile/Smartphone devices?).
- Facilitate speed and breadth of information dissemination.
- Provide one-to-one, one-to-many and many-to-many communication.
- Permit synchronous and asynchronous communication.
- Allow different levels of engagement.

**eLEARNING**

elarning or distance education is the use of electronic media and information technology to deliver education and training over a distance. eLearning has several forms and thus names depending upon the platforms used for the delivery of instructional contents: technology-enhanced learning, internet-based training, web-based training (WBT), virtual learning, virtual classroom, digital education, etc. These alternative names emphasize a variety of media employed for the delivery of education. The most recent term used for this type of education is MOOCs (Massive Online Open Courses) delivered over the Internet. Several prestigious educational institutions, like Columbia, New York and Harvard universities have started following this latest form of education, to which medical education would not be immune. 

**MOBILE LEARNING (mLearning)**

*Mobile learning denotes any sort of learning that happens when the learner is not at a fixed, predetermined location, or learning that happens when the learner takes advantage of the learning opportunities offered by mobile technologies.* ^9^ *Mobile* devices such as tablets and smartphones are enabling health organizations to provide learners with a whole new range of learning opportunities on health maintenance, balanced dieting, weight loss, management of chronic diseases, etc.  Mobile learning or mlearning, enables trainings to be:

- Accessed from anywhere where there is Internet or cellular service.
- Incorporated into blended learning programs in real-time.
- Used for real-time performance support.
- Delivered just-in-time.

By making computer-based training truly portable, mobile learning is enabling us to provide valuable training at the right place and the right time.

**VIRTUAL CLINICS/DOCTORS**

Virtual clinics are online clinics that provide 24-hour online access for patients to healthcare providers (clinicians) who can help diagnose and prescribe treatment, including prescriptions, of ordinary medical conditions. This type of healthcare delivery is relatively new and early indications suggest that it can improve the experience of care for patients and the health of populations, along with reducing per capita health care costs. Smart mobile devices now have an application called “Virtual Clinic” that allows doctors in the healthcare network to answer questions to patients in real time.

 Other smart mobile devices have “Virtual Doctor”^10^ application that helps provide a valuable support to users to improve their health conscious. It supports users to know some general information about their bodily symptoms, drugs and treatment, first aid, local hospitals, health information websites and provides information separately for men, woman and children.

## CONCLUSION

In summary, the emerging medical devices and technologies, especially the mobile phones and tiny Nano-sized sensors embedded in several electronic devices that enable sending and receiving information wirelessly, are changing the face of 21^st^ Century medical practice. The new technologies will allow remote monitoring of patients and their access to healthcare, health data collection, patient identification, medicine administration, medication compliance, information exchange with the providers and other patients, access to medical record, amongst several other benefits that would continue to accrue with the fast pace development of medical and allied health technologies.
